# Cannabis exposure and risk of testicular cancer: a systematic review and meta-analysis

**DOI:** 10.1186/s12885-015-1905-6

**Published:** 2015-11-11

**Authors:** J. Gurney, C. Shaw, J. Stanley, V. Signal, D. Sarfati

**Affiliations:** Department of Public Health, University of Otago, PO Box 7343, Wellington, New Zealand

**Keywords:** Testicular cancer, Testicular germ cell tumour, Cannabis, Marijuana, Marihuana, Seminoma, Non-seminoma

## Abstract

**Background:**

The aetiology of testicular cancer remains elusive. In this manuscript, we review the evidence regarding the association between cannabis use and testicular cancer development.

**Methods:**

In this systematic review and meta-analysis, we reviewed literature published between 1^st^ January 1980 and 13^th^ May 2015 and found three case–control studies that investigated the association between cannabis use and development of testicular germ cell tumours (TGCTs).

**Results/Conclusions:**

Using meta-analysis techniques, we observed that a) current, b) chronic, and c) frequent cannabis use is associated with the development of TGCT, when compared to never-use of the drug. The strongest association was found for non-seminoma development – for example, those using cannabis on at least a weekly basis had two and a half times greater odds of developing a non-seminoma TGCT compared those who never used cannabis (OR: 2.59, 95 % CI 1.60–4.19). We found inconclusive evidence regarding the relationship between cannabis use and the development of seminoma tumours. It must be noted that these observations were derived from three studies all conducted in the United States; and the majority of data collection occurred during the 1990’s.

**Electronic supplementary material:**

The online version of this article (doi:10.1186/s12885-015-1905-6) contains supplementary material, which is available to authorized users.

## Background

The cannabis plant has been ingested or inhaled by humans for more than 4000 years [1]. In the 2014 United Nations World Drug Report, it was estimated that some 178 million 15–64 year-olds worldwide use cannabis at least once per year – making it the most consumed illicit drug by a factor of five [2]. Substantial variability in the consumption of cannabis has been observed between (and within) populations – with prevalence considerably higher in the Americas, Europe and Oceania compared to Asia and Africa [2].

Testicular cancer is the most common cancer among young men, with peak incidence occurring between 15 and 40 years of age [3] and the highest rates of disease found among men who can trace their ancestry to Northern Europe [4]. Rates of testicular cancer appear to be increasing rapidly over time [5] – and yet the primary exposures involved in its aetiology remain poorly understood [6].

In recent years, at least three case–control studies reported associations between cannabis exposure and testicular germ cell tumour (TGCT) development [7–9]. A recent meta-analysis of these studies showed that those who used cannabis for longer than 10 years were 50 % more likely to develop testicular cancer than those who never used cannabis (summary odds ratio [OR]: 1.50, 95 % CI 1.08–2.09) [10]. However, this review was limited in two ways: firstly, it did not assess the quality of the case–control studies – an important step toward understanding potential sources of bias introduced by the authors; and secondly, it did not differentiate between seminoma and non-seminoma tumour types [10] – which is also important, since a) non-seminoma tumours are typically diagnosed seven [11] to ten [12] years earlier than seminoma tumours, and may differ in terms of risk factors; and b) each of the studies showed a stronger association for non-seminoma tumours than for seminoma tumours. This review aims to address these issues.

## Methods

In order to summarise the current evidence regarding the strength of association between cannabis exposure and testicular cancer, a systematic review and meta-analysis of the literature were undertaken. The review was performed in accordance with the Meta-analysis of Observational Studies in Epidemiology (MOOSE) guidelines [13].

### Search strategy

All articles published between 1^st^ Jan 1980 and 13^th^ May 2015 were eligible for inclusion. No limits were set in terms of language used or study design. A search of electronic databases was conducted on 13^th^ March 2015 using the following databases: Cinahl, Cochrane Library, Embase, Medline, ProQuest Central, ProQuest Dissertations and Theses, Scopus and Web of Science. Using a Boolean approach, we searched the electronic databases for any possible combination of the keywords listed in Table 1.

**Table 1:** List of exposure- and outcome-related keywords

Exposure-related keywords	Outcome-related keywords
Cannabi^a^	Cancer of the testi^a^
Marijuana	Seminoma^a^
Marihuana [29, 30]	Testi^a^ cancer
THC [31]	Testi^a^ carcinoma
Tetrahydrocannabinol [31]	Testi^a^ germ cell tumo(u)r
	Testi^a^ neoplasm
	Testi^a^ tumo(u)r

^a^indicates ‘explosion’ term

The reference lists of those studies which were considered eligible for inclusion (see below) were scanned for additional relevant studies. Two international experts in the field of testicular cancer and/or cancer epidemiology were contacted via email, and given a list of those studies which met our inclusion criteria. They were asked to identify any studies that had been missed by our search.

### Study inclusion

References were collected and logged in EndNote vX7.1 (Thomson Reuters, New York, U.S.A.). Duplicate records were removed prior to further analysis. Abstracts were screened by one reviewer (JG) to remove irrelevant studies, with a 10 % random sample of these verified by a second reviewer (VS). Any disagreements about inclusion were resolved by referral to a third reviewer (DS). The full text of all remaining papers was obtained and assessed by two reviewers (JG and VS) to identify those which met our inclusion criteria.

Studies included in the final analysis were those that reported associations between cannabis and testicular cancer. Studies were only included if data were provided from which summary associations (odds ratio or relative risks) and their 95 % confidence intervals could be calculated, or if these summary associations were provided by the authors themselves. All manuscripts that were considered relevant during the abstract screening process but ineligible for inclusion in our final analysis are listed in the supplementary material, along with justification for why they were ultimately excluded (Additional file 1).

### Data extraction

For each included study, one reviewer (JG) extracted meta-data, which was then verified by a second reviewer (VS). Meta-data included: study design, year of publication, location of study, sample size (cases/controls) sources of data, exclusion criteria, adjustment for confounding, methods of cannabis exposure measurement, and estimate of the association between outcome and exposure (Table 2).

**Table 2:** Papers included in meta-analysis of association between cannabis use and testicular cancer development, with study meta-data

Author	Year of publication	Study design	Study period	Year of data collection	Location of study	Sample size	Source of data	Exclusion criteria	Method of cannabis exposure measurement	Adjustment for confounding
Daling, et al. [9]	2009	CCS^c^	1999–2006	2006	Washington State, U.S.A.	369 cases/979 controls	Face-to-face interview	-Non-germ cell tumours -Choriocarcinoma -Age (<18 or >44) -No telephone -Non-English-speaking	Self-reported use of marijuana or hashish: -Ever-use -Age at first use -Duration of use -Frequency of use	-County^a^ -Age^a, b^ -Reference year^a, b^ -Alcohol use^b^ -Smoking status^b^ -Cryptorchidism^b^
Trabert et al. [7]	2011	CCS^c^	1990–1996	1996	Texas, U.S.A.	187 cases/148 controls	Self-completed questionnaire	-Non-germ-cell tumours -Age (<18 or >50) -Extragonadal tumours	Self-reported: -Ever-use -Duration of use -Frequency of use	-Age^a, b^ -Race^a, b^ -Alcohol use^b^ -Smoking status^b^ -Cryptorchidism^b^
Lacson et al. [8]	2012	CCS^c^	1986–1991	1987–1991	California, U.S.A.	163 cases/292 controls	Face-to-face interview	-Non-germ cell tumours -Age (<18 or >35) -Non-English-speaking -Born in U.S.A., Canada, Europe or Middle East	Self-reported: -Ever-use -Duration of use -Frequency of use	-Age^a^ -Race^a^ -Ethnicity^a^ -Neighbourhood^a^ -Cocaine use^b^ -Amyl Nitrate use^b^ -Cryptorchidism^b^ -Religiosity^b^ -Education^b^

^a^Adjustment for confounding achieved via control matching

^b^Adjustment for confounding achieved via inclusion as covariates in regression models

^c^ *CCS* case–control study

### Assessment of study quality

The assessment of study quality and potential for bias is an essential feature of any systematic review. However, there remains no gold standard measure of study quality for observational research. In the absence of such a gold standard, it has been recommended that any tools used to measure study quality should be as specific as possible to the given topic, and involve a simple checklist as opposed to a scale or score [14]. Given these factors, we assessed study quality and potential for bias using the criteria outlined in the Newcastle-Ottawa Quality Assessment Scale [15, 16], but did not determine a quality score [17]. Two reviewers (JG and JS) independently assessed study quality against these criteria, with disagreements resolved by referral to a third reviewer (DS).

### Statistical analysis

Adjusted odds ratios were extracted from each included study (along with their 95 % confidence intervals). We tested for evidence of heterogeneity between studies using both the *X*^2^ (p <0.1 indicating high inter-study heterogeneity) [18, 19] and I^2^ (0 % indicating no inter-study heterogeneity) [15, 19] tests. Using a random-effects model, we applied inverse-variance weighted methods for combining results across included studies to arrive at a final summary odds ratio (and associated 95 % confidence intervals) for the association between various levels of cannabis exposure and testicular cancer outcome (total and stratified by seminoma/non-seminoma tumours) [20]. This analysis was completed in Stata v11.2 using the *metan* function [21].

## Results

**Fig. 1:**
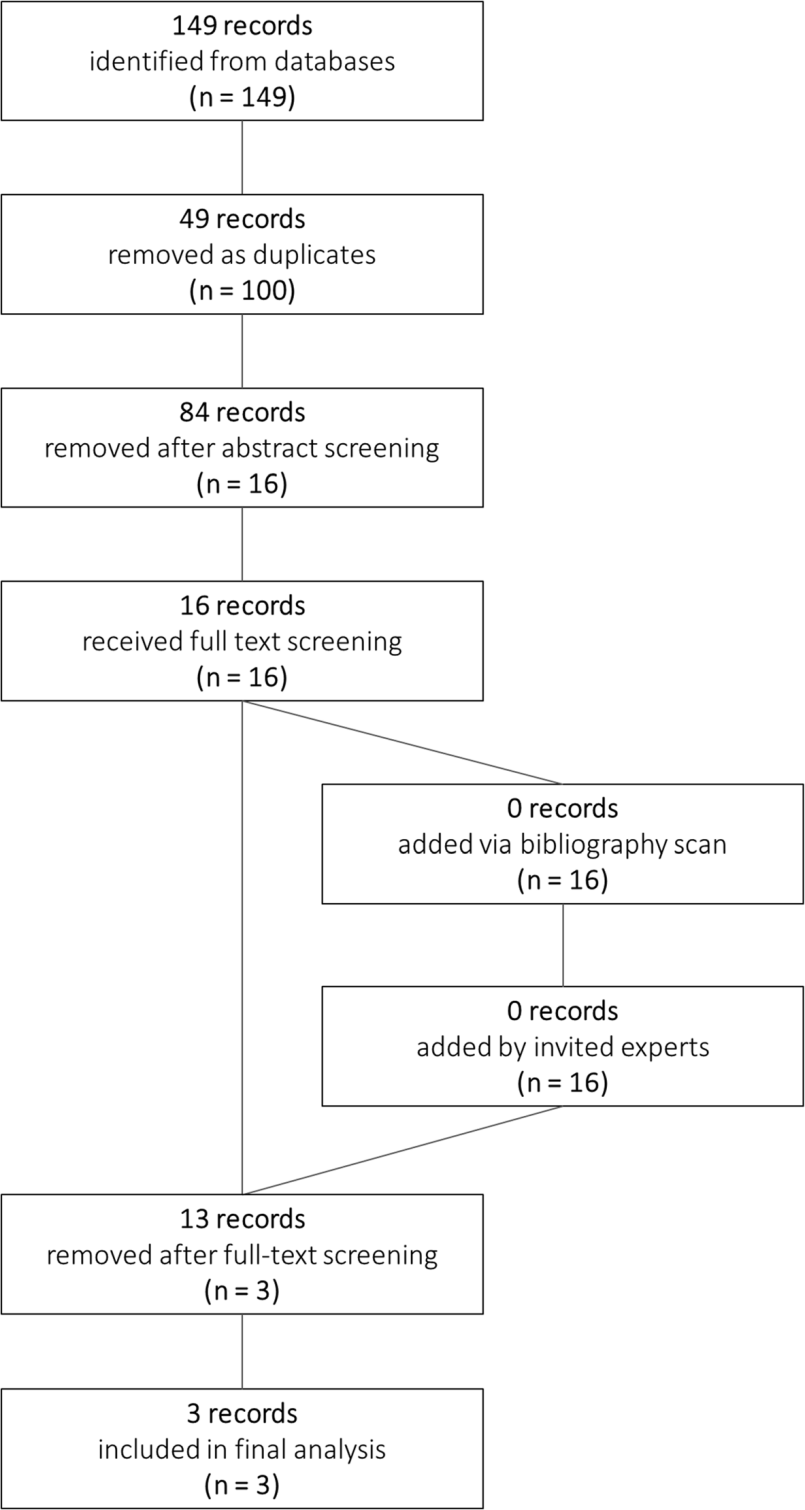
Flow chart of systematic review investigating association between cannabis exposure and testicular cancer development

Our search strategy resulted in the initial identification of 149 records. Forty-nine duplicate records were removed, leaving 100 unique studies. A further 84 records were removed as a result of abstract screening, which left a total 16 records for full-text screening to determine eligibility for analysis. No further records were added by scanning the reference list of the 16 records (Fig. 1).

Following full-text screening, 10 records were removed due to either lack of primary data or lack of relevance to the topic. A further 3 records were removed due to their primary data being formally published elsewhere – for example, primary data from a PhD thesis that was subsequently published in a peer-reviewed journal (Additional file 1). Following systematic review and exclusions, a total of 3 relevant case–control studies were found [7–9]. No cohort studies were found. Our invited experts both advised that they were unaware of any additional published studies of relevance to this review.

### Meta-data for included studies

Meta-data for each of the included studies are presented in Table 2. Each of the included studies were conducted in the United States, with the earliest recruitment occurring in 1986 [8] and the most recent occurring in 2006 [9]. A total of 719 cases with testicular germ cell tumours (TGCT) participated across the three studies, along with a total of 1419 controls. In all studies, cases were identified from local cancer registries and confirmed via review of pathology reports. In terms of histological type, two of the three studies separated the cohort into seminoma and non-seminoma sub-groups [8, 9], while the other study additionally separated non-seminoma tumours into non-seminoma and mixed-type sub-groups [7].

Controls were either randomly derived from the community [8, 9] or the friend of cases [7]. All three studies matched on age, while two of the three studies matched on region of residence [8, 9]. Two of the three studies also matched cases and controls on race and/or ethnicity [7, 8]. Two of the three studies [7, 9] frequency-matched controls to cases, while one study individually-matched controls to cases [8].

Cannabis exposure was measured using self-report, either via face-to-face interview [8, 9] or self-completed paper-based questionnaire [7]. Each of the included studies asked the participant to report ever-use of cannabis, the duration of use and the frequency of use – with one study also asking about age at first use [9].

With respect to adjustment for confounding – in addition to the covariates used to match controls to cases – all three studies adjusted for history of cryptorchidism, two of the studies additionally adjusted for use of alcohol and tobacco. [7, 9] One study also adjusted for other drug use (including amyl nitrate and cocaine), religiosity and education level [8].

### Assessment of study quality

The assessment of study quality against the Newcastle-Ottawa criteria is presented in Table 3. Case definition was adequate for all included studies, with registry records independently validated via review of pathology records. In terms of case representativeness, two of the included studies restricted their participants to those aged between 18 and 44–50 – with such practice being common in the testicular cancer context since a) the vast majority of cases occur within this age band, and b) it is thought that the aetiology of tumours that occur in younger or older populations differs to those that occur among this 18–50 year age group. One study (Lacson et al. [8]) further restricted their study groups to those aged 18–35.

**Table 3:** Assessment of the quality of studies included in current meta-analysis against the Newcastle-Ottawa criteria [16]

Author	Year	Adequacy of case definition	Representativeness of cases	Selection of controls	Definition of controls	Comparability of cases and controls	Ascertainment of exposure	Same ascertainment for cases and controls	Non-response rate	Author comment
Daling, et al. [9]	2009	Yes, with independent validation (1)	Consecutive or obviously representative series of cases (2)	Community controls (3)	No history of disease (4)	Cases and controls comparable (study controls for age and other factors) (5)	Interview not blinded to case/control status (6)	Yes (7)	Rate different (Response rate: cases 67.5 %/controls 43.3 %) (8)	Low response rate among controls (risk of selection bias). Largest study; strongest contributor to summary estimates
Trabert et al. [7]	2011	Yes, with independent validation (9)	Consecutive or obviously representative series of cases (10)	Community controls^a^ (11)	No history of disease (12)	Cases and controls comparable (study controls for age and other factors) (13)	Self- completed questionnaire (14)	Yes (15)	Rate different (Response rate: cases 38.2 %/controls 73.3 %) (16)	Low response rate among cases. Controls recruited as friends of cases (risk of selection bias)
Lacson et al. [8]	2012	Yes, with independent validation (17)	Consecutive or obviously representative series of cases (18)	Community controls (19)	No history of disease (20)	Cases and controls comparable (study controls for age and other factors) (21)	Interview not blinded to case/control status (22)	Yes (23)	Same rate for both groups (Response rate: cases 81.0 %/controls 78.7 %) (24)	Minimised to those aged 18–35 (limits representativeness)

Explanation of categorisations is presented in Additional file 2 alongside its corresponding number

^a^Controls derived from friends of cases

Each of the included studies derived their controls from the community, although one study used the friend of cases as controls [7], which may have reduced the representativeness of the control sample in that study. All controls had no history of testicular cancer. Each of the studies measured cannabis exposure in the same way (via self-report), although one asked about hashish exposure specifically as well as cannabis. For those studies in which person-to-person interview was conducted [8, 9], there is no record that interviewers were blinded as to the case/control status of the participant.

In order to maximise the comparability of cases and controls, each of the studies matched controls to cases – or adjusted in their regression modelling – for what could be considered the two strongest confounding variables (age and history of cryptorchidism).

Two of the included studies reported highly-differential response rates for cases and controls. One of these studies reported the highest response rate among cases (response rate: cases 67.5 %, controls 43.3 %) [9], while the other reported the highest response rate among controls (cases 38.2 %, controls 73.3 %) – the latter study deriving their controls from friends of cases [7]. The remaining study reported high and near-identical response rates between cases and controls (cases 81.0 %, controls 78.7 %) [8].

### Meta-analysis results

In terms of overall association, our meta-analysis was inconclusive regarding the association between ever-use of cannabis and development of TGCT (pooled odds ratio [OR], ever-use compared with never use): 1.19, 95 % CI 0.72–1.95), and for the association of former use with TGCT (OR: 1.54, 95 % CI 0.84–2.85). We observed that current use of cannabis increased the odds of TGCT development by 62 % (OR: 1.62, 95 % CI 1.13–2.31). Frequency of cannabis was associated with TGCT development, with weekly (or greater) use appearing to nearly doubling the odds of TGCT development (OR: 1.92, 95 % CI 1.35–2.72). There was also evidence of an association between the duration of cannabis use (> = 10 years vs. never use) and TGCT development (OR: 1.50, 95 % CI 1.08–2.09).

**Fig. 2:**
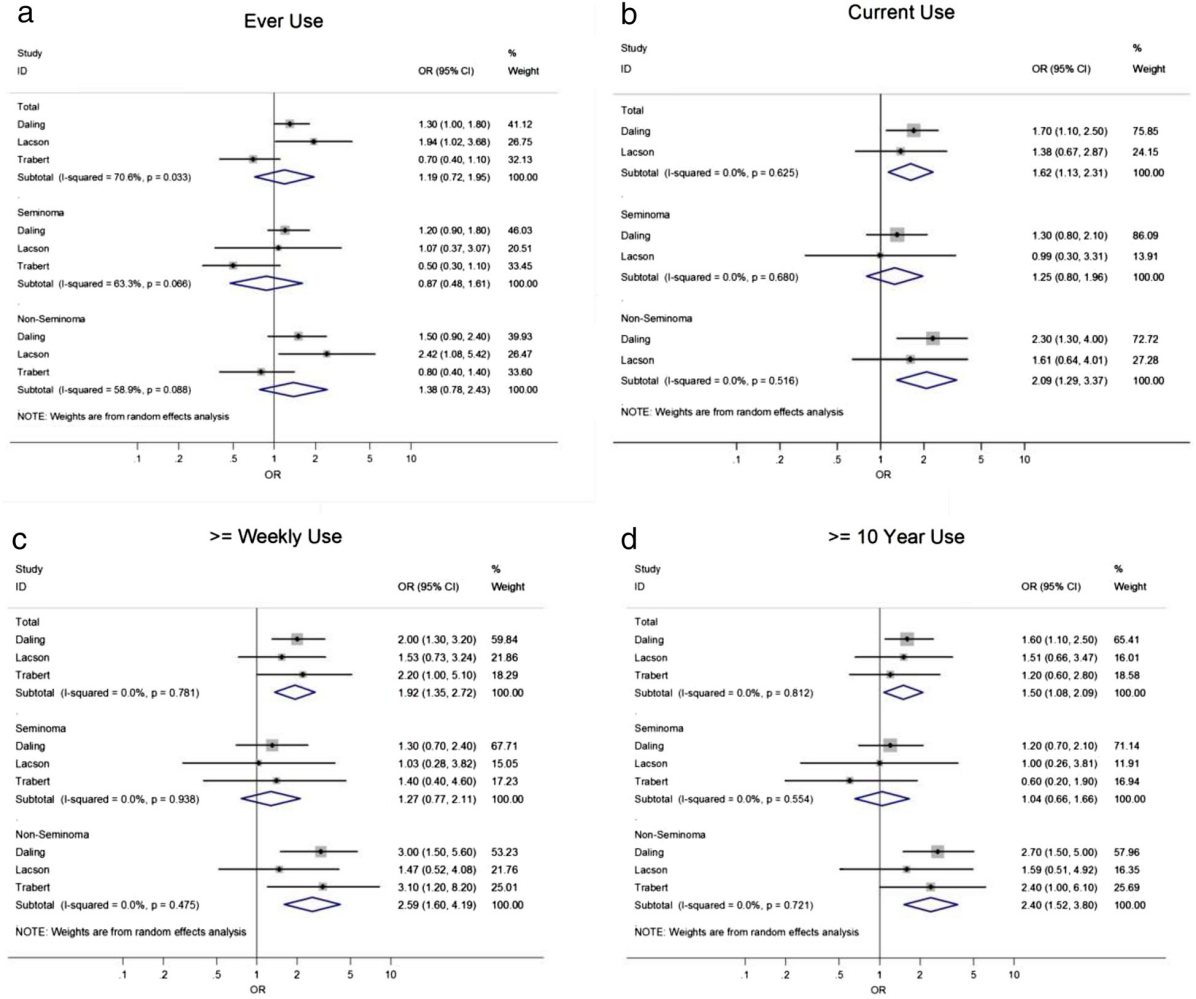
Forest plots – with odds ratios and heterogeneity statistics – for **a** ever-use, **b** current use, **c** > = weekly use, and **d** > =10 years of use. (Total = all histological types)

There was insufficient evidence to conclude that cannabis use was associated with seminoma development (Fig. 2). However, there was evidence of an association between cannabis use and non-seminoma development – with current use more than doubling the odds of tumour development (OR: 2.09, 95 % CI 1.29–3.37). Frequency of use was also strongly associated with non-seminoma development, with those using cannabis on at least a weekly basis having two and a half times greater odds of tumour development compared those who never used cannabis (OR: 2.59, 95 % CI 1.60–4.19). Finally, those who had used cannabis for at least 10 years had nearly two and half times the odds of non-seminoma development compared to never-users (OR: 2.40, 95 % CI 1.52–3.80).

In terms of heterogeneity, a high level of agreement between studies was found – with I^2^ values of 0 % observed for most exposure variables (Fig. 2b-d). A notable exception was the ever-use variable (Fig. 2a), for which I^2^ values ranged between 59 % (non-seminoma tumour type) and 71 % (combined tumour types).

## Discussion

The results of this review show that current use of cannabis (pooled summary OR: 1.62, 95 % CI 1.13–2.31), using cannabis on at least a weekly basis (OR: 1.92, 95 % CI 1.35–2.72) and long duration (>10 years) of cannabis use (OR: 1.50, 95 % CI 1.08–2.09) are all associated with an increased risk of development of TGCT overall, and even more strongly with non-seminoma tumours specifically. There was insufficient evidence to conclude that there is a relationship between seminoma tumours and cannabis use.

Thus, our meta-analyses suggest that a strong association exists between TGCT development and current, chronic and/or frequent cannabis use – particularly non-seminoma development –when compared to those who have never used cannabis.

### Biological plausibility of cannabis in testicular carcinogenesis

The primary psychoactive component of the cannabis plant – delta-9-tetrahydrocannabinol, or *THC* – stimulates neural cannabinoid receptors, mimicking the action of endogenous cannabanoids (termed *endocannabanoids*). The position of these cannabinoid receptors in the basal ganglia, hippocampus, cerebellum and neocortex explains the common neurophysiological effects of cannabis ingestion; however, these receptors are also expressed in peripheral locations, including the testis [22].

The biological plausibility of the link between cannabis exposure and testicular cancer is thought to be related to disruptions to the hypothalamic–pituitary–testicular axis – an endocrine feedback system which, among other actions, assists with spermatogenesis [23]. It is thought that cannabis exposure – and subsequent stimulation of cannabinoid receptors – disrupts normal hormone regulation and testicular function, and that this disruption leads to carcinogenesis [23]. However, evidence regarding the association between regulation of normal testicular function and tumour development remains inconclusive; and given the complex and multifaceted influence of cannabinoid receptor stimulation on biological processes [9], the path from cannabis exposure to testicular carcinogenesis remains unclear.

### Timing of cannabis exposure

The observation of an association between cannabis use and non-seminoma TGCT development – but not seminoma development – is intriguing. As discussed by Skeldon and Goldenberg [23], this association directs our attention to puberty (rather than later in life) as the key point of exposure. Non-seminoma tumours are typically diagnosed seven [11] to ten [12] years earlier than seminoma tumours. Interestingly, one study included in the current review that asked cases and controls about the timing of their first cannabis use showed that those who first-used before the age of 18 years were substantially more likely to develop a non-seminoma TGCT compared to never-users (adjusted OR: 2.80, 95 % CI 1.60–5.10), but that those aged 18 or older were not (OR: 1.30, 95 % CI 0.60–3.20) [9]. This may suggest that any carcinogenic disruption of interest to the hypothalamic–pituitary–testicular axis occurs during (or before) puberty [23]; however it is also possible that early initiation of cannabis exposure is a marker of other mediating factors, such as duration and frequency of cannabis use later in life. Another possibility is that since those cases that developed non-seminoma tumours were younger at the time of data collection than those who developed seminoma tumours, they may have been more likely to either recall or report marijuana use. Such a scenario would have the effect of exaggerating the association between cannabis use and non-seminoma development. However, it should be noted that this exaggeration would only occur if the age-matched controls who participated in these studies were not affected by this pattern of differential reporting by age – in other words, if the cannabis use reported by controls was accurate. This is an area that warrants further exploration.

An as-yet unexplored concept regarding the timing of cannabis exposure is the period during prenatal and early childhood development. Best current evidence suggests that TC predisposition is determined prenatally; thus, it is possible that those who positively identify as current, chronic cannabis users are also more likely to have been exposed to cannabis during perinatal and/or early childhood development. In other words, it is possible that primary cannabis use could be a proxy for second-hand exposure to cannabis during the prenatal and/or early childhood period. Such exposure would be congruent with a pre-adulthood disruption to the hypothalamic-pituitary-testicular axis, albeit via a secondary rather than primary source. However this association remains speculative and further research is required regarding the role of non-primary exposure to cannabis during the prenatal and early childhood period as a risk factor for the development of TGCT.

### Strengths and weaknesses of included studies

The three case–control studies examined for this review had strengths in a number of areas; however, each of the studies had acknowledged weaknesses, one of these being the ascertainment of cannabis exposure.

For all three studies, exposure to cannabis was measured using self-report – either during a face-to-face interview [8, 9] or on a written questionnaire [7]. According to the Newcastle-Ottawa Scale, one of the optimum mechanisms to measure exposure – and ostensibly minimise risk of information bias – is via a structured interview, where the interviewer is blinded to the case/control status of the participant. There is no record in any of the included studies that the interviewers were blinded to the status of the participant. The importance of this is that we do not whether (and to what extent) the association between cannabis exposure and TC was affected by interviewer bias (i.e., the interviewer knowing the case/control status of the participant, and inadvertently leading the participant toward certain answers). However, it would seem unlikely that interviewer bias could explain all or even some of the observed associations between cannabis use and TGCT development; for example, it is difficult to imagine a scenario where knowledge of case/control status would cause interviewers to inadvertently lead those with non-seminoma tumours toward one response, and those with seminoma tumours to another.

In the presence of an association between current cannabis use and testicular cancer development, it would also be desirable to validate self-reported current (or non-current) use via an appropriate specimen-based test. [24] However the absence of a valid and easily-obtainable biomarker that does not involve the participant providing a urine sample may render such an approach untenable. It is possible that the use of self-report only will underestimate current use of cannabis [24–26]; however there is also some evidence that self-report is an efficacious means of classifying current (or recent) exposure to cannabis among men of similar age to participants of the three included studies [27].

If the cases and controls are equally likely to either under- or over-report cannabis exposure, then the impact on the observed association between cannabis use and TGCT development would likely be to attenuate it. However, if TGCT cases are more likely to report/recall cannabis use than controls – because of concern that cannabis or similar exposures might be a cause of their cancer, or a similar reason – then this may serve to exaggerate the reported association away from the null. Of course, it is entirely possible that the same exaggeration could occur if cases reported their use accurately, but controls under-reported their use.

The second major weakness for two of the three included studies was low and differential response rates. In one study, the response rate was substantially lower among the controls than the cases [9]. If the reported cannabis use was different among those controls who responded compared with those who did not, and if the same differential is not present for the cases who responded and cases who did not respond, this will result in biased OR. For example, if the controls who responded had lower rates of cannabis use than non-responding controls, this will lead to an overestimate of the cannabis-TGCT association. Unusually in a second study, the control group had a substantially higher response rate than the case group [7]. In this study, the controls were friends of the cases, which may explain their willingness to participate in the study. However it is not clear why the response rate among cases was so low. For this latter study, it may be reasonable to assume that cannabis use might have been more similar between cases and controls than if unrelated controls were used. If this is true, we might expect that the ORs in this study would be biased towards the null. Reassuringly, the results of all three studies were reasonably consistent despite the different potential sources of selection bias.

Finally, when considering the role of cannabis in the development of testicular cancer we must also consider the likely pervasiveness of this exposure. For example, it was estimated in the World Drug Report that 12 % of U.S. residents aged 12 or older had used cannabis in 2012 [2], with 36 % of U.S. college students reported to have used the drug in 2013 [28]. Given this pervasiveness among young adults, it is likely that ‘ever-use’ will include many individuals with very low exposure to cannabis – meaning that ever-use is unlikely to be a true measure of meaningful cannabis exposure.

It is also worth noting that of all the exposure variables included in our meta-analysis, the greatest heterogeneity between studies was observed for the ever-use variable (I^2^ > 50 %). The source of this heterogeneity is obscure and likely to be multifaceted – but could plausibly be due to heterogeneity between study populations in terms of a) pervasiveness of cannabis ever-use and/or b) willingness to report it. For example, fewer controls in the study by Trabert et al. (55 %) [7] reported ever-use of cannabis compared to the study by Daling et al. (68 %) [9].

## Conclusions

Using meta-analysis of published studies, we observed that a) current, b) chronic, and c) frequent cannabis use is associated with the development of TGCT – particularly non-seminoma TGCT – at least when compared to never-use of the drug. We found inconclusive evidence regarding the relationship between ever- and former-use of cannabis and TGCT development. However, it must be noted that these observations were derived from only three published studies; that these studies were all conducted in the United States; and the majority of data collection occurred during the 1990’s.

## Additional files

Additional file 1:
**List of papers excluded from the current meta-analysis following full-text screening, and the reason for their exclusion.** (DOCX 17 kb) Additional file 2:
**Detailed critique of manuscripts included in the current meta-analysis against Newcastle-Ottawa Scale criteria.** (DOCX 19 kb)

## Abbreviations

MOOSE: Meta-analysis of observational studies in epidemiology
OR: Odds ratio
TC: Testicular cancer
TGCT: Testicular germ cell tumour

## References

[1] Murray RM, Morrison PD, Henquet C, Forti MD (2007). Cannabis, the mind and society: the hash realities. Nat Rev Neurosci.

[2] UNODC (2014). World drug report.

[3] Winter C, Albers P (2011). Testicular germ cell tumors: pathogenesis, diagnosis and treatment. Nat Rev Endocrinol.

[4] McGlynn KA, Cook MB (2009). The epidemiology of testicular cancer. Male reproductive cancers: epidemiology, pathology and genetics.

[5] Nigam M, Aschebrook-Kilfoy B, Shikanov S, Eggener S (2014). Increasing incidence of testicular cancer in the United States and Europe between 1992 and 2009. World J Urol.

[6] McGlynn KA, Trabert B (2012). Adolescent and adult risk factors for testicular cancer. Nat Rev Urol.

[7] Trabert B, Sigurdson AJ, Sweeney AM, Strom SS, McGlynn KA (2011). Marijuana use and testicular germ cell tumors. Cancer.

[8] Lacson JCA, Carroll JD, Tuazon E, Castelao EJ, Bernstein L, Cortessis VK (2012). Population-based case–control study of recreational drug use and testis cancer risk confirms an association between marijuana use and nonseminoma risk. Cancer.

[9] Daling JR, Doody DR, Sun X, Trabert BL, Weiss NS, Chen C (2009). Association of marijuana use and the incidence of testicular germ cell tumors. Cancer.

[10] Huang Y-HJ, Zhang Z-F, Tashkin DP, Feng B, Straif K, Hashibe M (2015). An epidemiologic review of marijuana and cancer: an update. Cancer Epidemiol Biomarkers Prev.

[11] Baade P, Carrière P, Fritschi L (2008). Trends in testicular germ cell cancer incidence in Australia. Cancer Causes Control.

[12] Huyghe E, Matsuda T, Thonneau P (2003). Increasing incidence of testicular cancer worldwide: a review. J Urol.

[13] Stroup D, Berlin J, Morton S, Olkin I, Williamson G, Rennie D (2000). Meta-analysis of observational studies in epidemiology: a proposal for reporting. JAMA.

[14] Sanderson S, Tatt ID, Higgins JPT (2007). Tools for assessing quality and susceptibility to bias in observational studies in epidemiology: a systematic review and annotated bibliography. Int J Epidemiol.

[15] Alam SS, Cantwell MM, Cardwell CR, Cook MB, Murray LJ (2010). Maternal body mass index and risk of testicular cancer in male offspring: a systematic review and meta-analysis. Cancer Epidemiol.

[16] Wells G, Shea B, O’Connell D, Peterson J, Welch V, Losos M, et al. Quality assessment scales for observational studies. Ottawa, Canada: Ottawa Health Research Institute; 2004.

[17] Burkey MD, Feirman S, Wang H, Choudhury SR, Grover S, Johnston FM (2014). The association between smokeless tobacco use and pancreatic adenocarcinoma: a systematic review. Cancer Epidemiol.

[18] Kirkwood BR, Sterne JA (2003). Essential medical statistics.

[19] Michos A, Xue F, Michels KB (2007). Birth weight and the risk of testicular cancer: a meta-analysis. Int J Cancer.

[20] Lip SZL, Murchison LED, Cullis PS, Govan L, Carachi R (2013). A meta-analysis of the risk of boys with isolated cryptorchidism developing testicular cancer in later life. Arch Dis Child.

[21] Sterne JA, Bradburn MJ, Egger M. Meta-analysis in stata. Systematic reviews in health care: meta-analysis in context. 2nd ed. London, United Kingdom: Wiley; 2006. p. 347–69.

[22] Guzmán M (2003). Cannabinoids: potential anticancer agents. Nat Rev Cancer.

[23] Skeldon SC, Goldenberg SL (2014). Urological complications of illicit drug use. Nat Rev Urol.

[24] Buchan BJ, Dennis ML, Tims FM, Diamond GS (2002). Cannabis use: consistency and validity of self-report, on-site urine testing and laboratory testing. Addiction.

[25] de Beaurepaire R, Lukasiewicz M, Beauverie P, Castéra S, Dagorne O, Espaze R (2007). Comparison of self-reports and biological measures for alcohol, tobacco, and illicit drugs consumption in psychiatric inpatients. Eur Psychiatry.

[26] Jain R, Quraishi R, Majumder P, Pattanayak RD (2013). Comparison of self-report and biological measures for alcohol, tobacco and illicit drug use in consecutive alcohol-dependent patients visiting a tertiary care centre. J Subst Use.

[27] Mayet A, Esvan M, Marimoutou C, Haus-Cheymol R, Verret C, Ollivier L (2013). The accuracy of self-reported data concerning recent cannabis use in the French armed forces. Eur J Public Health.

[28] Johnston LD, O’Malley PM, Bachman JG, Schulenberg JE, Miech RA (2014). Monitoring the future - national survey results on drug use, 1975–2013: Volume 2, College students and adults ages 19–55.

[29] Kedzior KK, Laeber LT (2014). A positive association between anxiety disorders and cannabis use or cannabis use disorders in the general population- a meta-analysis of 31 studies. BMC Psychiatry.

[30] Lev-Ran S, Roerecke M, Le Foll B, George TP, McKenzie K, Rehm J (2014). The association between cannabis use and depression: a systematic review and meta-analysis of longitudinal studies. Psychol Med.

[31] Szoke A, Galliot AM, Richard JR, Ferchiou A, Baudin G, Leboyer M (2014). Association between cannabis use and schizotypal dimensions - a meta-analysis of cross-sectional studies. Psychiatry Res.

