# Supplementary material for: Cannabis exposure and risk of testicular cancer: a systematic review and meta-analysis
Source: BMC Cancer. 2015 Nov 11;15:897. doi: 10.1186/s12885-015-1905-6 (PMC4642772; doi:10.1186/s12885-015-1905-6)
Supplement: Additional file 1: — List of papers excluded from the current meta-analysis following full-text screening, and the reason for their exclusion. (DOCX 17 kb) [file 12885_2015_1905_MOESM1_ESM.docx]

**Additional File 1:** List of papers excluded from the current meta-analysis following full-text screening, and the reason for their exclusion.

| **Author** | **Year** | **Title** | **Reason for exclusion** | **Reference** |
| --- | --- | --- | --- | --- |
| Castelao, J.E. | 1998 | Population-based case-control study of testicular cancer in young men (PhD thesis) | Results of same study presented in Lacson et al., 2012 | Castelao, J. E. (1998). Population-based case-control study of testicular cancer in young men. (9912650 Ph.D.), University of California, Los Angeles, Ann Arbor. |
| Hall, W. & Degenhardt, L. | 2009 | Adverse health effects of non-medical cannabis use | No primary data / not relevant to testicular cancer | Hall, W., & Degenhardt, L. (2009). Adverse health effects of non-medical cannabis use. Lancet, 374(9698), 1383-1391. |
| Hall, W. & Degenhardt, L. | 2014 | The adverse health effects of chronic cannabis use | No primary data / not relevant to testicular cancer | Hall, W., & Degenhardt, L. (2014). The adverse health effects of chronic cannabis use. Drug Testing and Analysis, 6(1-2), 39-45. doi: 10.1002/dta.1506 |
| Huang, Y-H J, et al. | 2015 | An epidemiologic review of marijuana and cancer: an update | No primary data | Huang, Y. H. J., Zhang, Z. F., Tashkin, D. P., Feng, B., Straif, K., & Hashibe, M. (2015). An epidemiologic review of marijuana and cancer: An update. Cancer Epidemiology Biomarkers and Prevention, 24(1), 15-31. |
| Lacson, J.C., et al. | 2013 | Potential impact of age at first marijuana use on the development of nonseminomatous testicular germ cell tumors | No primary data | Lacson, J. C. A., Bernstein, L., & Cortessis, V. K. (2013). Potential impact of age at first marijuana use on the development of nonseminomatous testicular germ cell tumors. Cancer, 119(6), 1284-1285. |
| Marcus, D.M., et al. | 2013 | Population-based case-control study of recreational drug use and testis cancer risk confirms an association between marijuana use and nonseminoma risk (Letter to editor) | No primary data | Marcus, D. M., Jani, A. B., & Rossi, P. J. (2013). Population-based case-control study of recreational drug use and testis cancer risk confirms an association between marijuana use and nonseminoma risk. Cancer, 119(6), 1284. |
| McGlynn, K.A. & Trabert, B. | 2012 | Adolescent and adult risk factors for testicular cancer | No primary data | McGlynn, K. A., & Trabert, B. (2012). Adolescent and adult risk factors for testicular cancer. Nature Reviews Urology, 9(6), 339-349. |
| Meeks, J.J., et al. | 2012 | Environmental toxicology of testicular cancer | No primary data | Meeks, J. J., Sheinfeld, J., & Eggener, S. E. (2012). Environmental toxicology of testicular cancer. Urologic Oncology: Seminars and Original Investigations, 30(2), 212-215. |
| Myung, N.K. | 2014 | Risk factors of testicular germ cell tumor (Masters thesis) | No primary data | Myung, N. K. (2014). Risk factors of testicular germ cell tumor. (1560563 M.S.), University of Southern California, Ann Arbor. |
| Richards, L. | 2009 | Testicular cancer: Marijuana use linked with testicular cancer. | No primary data | Richards, L. (2009). Testicular cancer: Marijuana use linked with testicular cancer. Nature Reviews Urology, 6(4), 180. |
| Sigurdson, A.J. | 1997 | Hormonal risk factors and testicular cancer (PhD thesis) | Results of same study presented in Trabert et al., 2011 | Sigurdson, A. J. (1997). Hormonal risk factors and testicular cancer. (9809551 Ph.D.), The University of Texas Health Sciences Center at Houston School of Public Health. |
| Smith, N.D. | 2012 | Environmental exposures and genitourinary malignancies | No primary data | Smith, N. D. (2012). Environmental exposures and genitourinary malignancies. Urologic Oncology: Seminars and Original Investigations, 30(2), 196-198. |
| Trabert, B., et al. | 2010 | Marijuana use and testicular germ cell tumors (Conference proceeding) | Results of same study presented in Trabert et al., 2011 | Trabert, B., Sigurdson, A. J., Sweeney, A. M., Strom, S. S., & McGlynn, K. A. (2010). Marijuana use and testicular germ cell tumors. 101st Annual Meeting of the American Association for Cancer Research, AACR 2010 Washington, DC United States. |
