# Supplementary material for: Cannabis exposure and risk of testicular cancer: a systematic review and meta-analysis
Source: BMC Cancer. 2015 Nov 11;15:897. doi: 10.1186/s12885-015-1905-6 (PMC4642772; doi:10.1186/s12885-015-1905-6)
Supplement: Additional file 2: — Detailed critique of manuscripts included in the current meta-analysis against Newcastle-Ottawa Scale criteria. (DOCX 19 kb) [file 12885_2015_1905_MOESM2_ESM.docx]

**Additional File 2:** Detailed critique of manuscripts included in the current meta-analysis against Newcastle-Ottawa Scale criteria.

***Daling et al.:***

1. Daling et al. derived cases in the first instance from a cancer registry (CSS; part of the SEER programme). They then asked (although they did not state how this was achieved, i.e. by letter, phone call or face-to-face interview) with each patient’s follow-up physician, and asked them if there was any reason why the man should not be included in the study. The absence of any such reason, an introductory letter and invitation to participate was sent to the patient.
2. Daling et al. minimised their cohort to those aged 18-44, thus limiting the representativeness of their cohort to those TC patients who are within this age band. However, such practice is common in TC research since it is thought that the aetiology of disease that occurs during childhood or later in life differs to that which occurs among those aged 18-50.
3. Controls in this study were identified from the community, via random telephone digit-dialling.
4. Controls in this study had not history of TC.
5. The study matched controls based on age, arguably the strongest confounder in the context of a TC case-control study. They also match controls on region of residence and reference year, and then adjust for other potential confounders (alcohol consumption, smoking status and history of cryptorchidism) in their regression models.
6. The exposure (i.e. cannabis use) was ascertained via interview. There is no record that the interviewer was blinded to the outcome status of the participant (i.e. whether they were a case or control).
7. Exposure to cannabis was measured in the same way for cases and controls (i.e. self-report).
8. Information on control participation is available in a separate manuscript that uses data from the same case-control study (Biggs et al., 2008).The response rate among controls (43.3%) was a third lower than the response rate among cases (67.5%).

***Trabert et al.:***

1. Trabert et al. derived cases in the first instance from a series of men seen at a major medical centre with a diagnosis of testicular cancer (from The University of Texas M. D. Anderson Cancer Center). Pathology reports for each case were then reviewed, and cases grouped according to tumour histology.
2. Trabert et al. minimised their cohort to those aged 18-50, thus limiting the representativeness of their cohort to those TC patients who are within this age band. However, such practice is common in TC research since it is thought that the aetiology of disease that occurs during childhood or later in life differs to that which occurs among those aged 18-50.
3. The controls in this study were friends – of the same race and similar age – of the cases. The results presented by Trabert et al. were for unmatched analyses – derived from unconditional logistic regression models.
4. While it is not directly reported in Trabert et al., another manuscript describing the methods used in their case-control study (Sigurdson et al., 1999) reported that controls had no history of cancer.
5. Trabert et al. attempted to match controls based on age by asking cases to refer friends of the same race and similar age to them into the study (to act as controls). However they reported that cases tended to refer friends who were older than them, and so decided to additionally adjust for age (and race) in their regression models. They also adjusted for history of cryptorchidism, alcohol use and cigarette smoking in their models.
6. The exposure (i.e. cannabis use) was ascertained via self-report (self-administered questionnaire).
7. Exposure to cannabis was measured in the same way for cases and controls (i.e. self-administered questionnaire).
8. The response rate for this study was found in Sigurdson et al. (1999). As described in that manuscript, 490 potential cases were approached to participate in the study, of whom 187 agreed (38.2%) – while 202 healthy controls were approached to participate, of whom 73.3% agreed to participate.

***Lacson et al.:***

1. Lacson et al. derived cases in the first instance from the Los Angeles Cancer Surveillance Program, which is a population-based cancer registry that is part of the SEER network. The pathology reports for each case were then reviewed, and cases grouped according to tumour histology.
2. Lacson et al. limited their cohort to those aged 18-35. As described for both Daling et al. and Trabert et al., it is common practice to limit investigations in the testicular cancer context – observational or otherwise – to those aged 18-50, under the assumption that the aetiology of disease that occurs during childhood or later in life differs to that which occurs among those aged 18-50. However, Lacson et al. restricted the upper-ranger of their cohort to 35 years of age – approximately the median age of testicular cancer diagnosis (Gurney et al., 2015). By minimising their cohort to those aged 18-35, Lacson et al. effectively reduced the representativeness of their observations to those within this age band – since we remain uncertain regarding the timing of cannabis exposure and how this relates to testicular cancer development.
3. Controls were derived from the community, by ‘canvassing’ housing units for eligible controls. The methods used during this canvassing were not detailed in the manuscript.
4. Controls were ‘unaffected’ by testicular cancer. A self-reported history of urogenital disorders was also incorporated into the questionnaire.
5. The study matched controls based on age, arguably the strongest confounder in the context of a TC case-control study. They also match controls on race and ethnicity (with no definition given regarding differentiation between these variables), as well as neighbourhood of residence. They also adjusted for history of cryptorchidism, religiosity and education level in their regression models – confounders identified using stepwise regression methods, whereby those covariates which affected the point estimate by greater than 10% were considered confounders and thus included in the model. Use of other recreational drugs (cocaine and amyl nitrate) were also included as covariates in the models.
6. The exposure (i.e. cannabis use) was ascertained via interview. There is no record that the interviewer was blinded to the outcome status of the participant (i.e. whether they were a case or control).Exposure to cannabis was measured in the same way for cases and controls (i.e. self-report during interview).
7. Lacson et al. were able to report near-identical response rates among eligible cases (163 out of 201) and controls (292 out of 371) who were asked to participate in the study.
